# The 752delG26 mutation in the RFXANK gene associated with major histocompatibility complex class II deficiency: evidence for a founder effect in the Moroccan population

**Published:** 2011-01-10

**Authors:** H Naamane, F Ailal, O Abidi, L Jeddane, J Najib, A Barakat, AA Bousfiha

**Affiliations:** 1 Laboratoire d'Immunologie, Institut Pasteur, Casablanca, Morocco; 2 Unité d'Immunologie Clinique, Service de Pédiatrie 1, CHU Ibn Rochd, Casablanca, Morocco; 3 Laboratoire d'Immunologie, CHU Ibn rochd, Casablanca, Morocco; 4 Laboratoire de génétique humaine, Institut Pasteur, Casablanca, Morocco

## 

Major histocompatibility complex class II plays a key role in the immune response, by presenting processed antigens to CD4+ lymphocytes. Major histocompatibility complex class II expression is controlled at the transcriptional level by at least four trans-acting genes: CIITA, RFXANK, RFX5 and RFXAP. Defects in these regulatory genes cause MHC class II immunodeficiency, which is frequent in North Africa. The aim of this study was to describe the immunological and molecular characteristics of ten unrelated Moroccan patients with MHC class II deficiency. Immunological examinations revealed a lack of expression of MHC class II molecules at the surface of peripheral blood mononuclear cells, low CD4+ T lymphocyte counts and variable serum immunoglobulin (IgG, IgM and IgA) levels. In addition, no MHC class II (HLA DR) expression was observed on lymphoblasts.

The molecular analysis identified the same homozygous 752delG26 mutation in the RFXANK genes of all patients. This finding confirms the association between the high frequency of the combined immunodeficiency and the defect in MHC class II expression and provides strong evidence for a founder effect of the 752delG26 mutation in the North African population. These findings should facilitate the establishment of molecular diagnosis and improve genetic counseling for affected Moroccan families.

